# TNF-Alpha Pathway Alternation Predicts Survival of Immune Checkpoint Inhibitors in Non-Small Cell Lung Cancer

**DOI:** 10.3389/fimmu.2021.667875

**Published:** 2021-09-16

**Authors:** Anqi Lin, Hongman Zhang, Hui Meng, Ze Deng, Tianqi Gu, Peng Luo, Jian Zhang

**Affiliations:** Department of Oncology, Zhujiang Hospital, Southern Medical University, Guangzhou, China

**Keywords:** TNFα, NSCLC, ICIs, biomarker, tumor microenvironment

## Abstract

Translational research on immune checkpoint inhibitors (ICIs) has been underway. However, in the unselected population, only a few patients benefit from ICIs. Therefore, screening predictive markers of ICI efficacy has become the current focus of attention. We collected mutation and clinical data from an ICI-treated non-small cell lung cancer (NSCLC) cohort. Then, a univariate Cox regression model was used to analyze the relationship between tumor necrosis factor α signaling mutated (TNFα-MT) and the prognosis of immunotherapy for NSCLC. We retrospectively collected 36 NSCLC patients (local-cohort) from the Zhujiang Hospital of Southern Medical University and performed whole-exome sequencing (WES). The expression and mutation data of The Cancer Genome Atlas (TCGA)-NSCLC cohort were used to explore the association between TNFα-MT and the immune microenvironment. A local cohort was used to validate the association between TNFα-MT and immunogenicity. TNFα-MT was associated with significantly prolonged overall survival (OS) in NSCLC patients after receiving immunotherapy. Additionally, TNFα-MT is related to high immunogenicity (tumor mutational burden, neoantigen load, and DNA damage response signaling mutations) and enrichment of infiltrating immune cells. These results suggest that TNFα-MT may serve as a potential clinical biomarker for NSCLC patients receiving ICIs.

## Introduction

Lung cancer is a disease with very high morbidity and mortality among all malignant tumors in the world (1–3). In the past decades, the 5-year overall survival (OS) rate of patients with advanced lung cancer has been only 5% (4). Histologically, lung cancer is mainly divided into non-small cell lung cancer (NSCLC) and small cell lung cancer (SCLC). NSCLC accounts for more than 85% of all lung cancer cases and is the most common histological subtype (5, 6). The current main treatment plan for NSCLC is a comprehensive treatment based on surgery, radiotherapy, chemotherapy and molecular targeted therapy.

With the advent of the era of precision medicine, targeting programmed cell death protein 1/programmed cell death ligand-1 (PD-1/PD-L1) and cytotoxic T-lymphocyte-associated antigen 4 (CTLA-4) has revolutionized cancer treatment and improved the long-term survival rate of patients with advanced NSCLC (7–10). However, growing evidence have shown that anti-PD-1/PD-L1 monotherapy produces long-lasting (>6 months) clinical benefits for only a small number of patients (15% to 19.4% in phase I/II clinical trials) (7–9, 11); thus, biomarkers with high specificity and detection rates are needed to predict the efficacy of PD-1/PD-L1 immune checkpoint inhibitors (ICIs).

Currently, PD-L1 expression is approved as a biomarker for immunotherapy (12, 13); PD-L1 is an inducible and dynamic biomarker for ICI treatment for multiple cancer types. Additionally, PD-L1 is expressed not only on the surface of tumor cells but also on immune cells in tumor tissues, and its expression can be affected by cell growth mediator such as IFNγ. Therefore, the expression is still an imperfect biomarker for predicting the efficacy of anti-PD-1/PD-L1 therapy in NSCLC (14–16). Tumor mutational burden (TMB) can also be used as a marker for determining the efficacy of immunotherapy. However, these markers also have some limitations (17–19). For example, it is difficult to standardize the “high” and “low” cut-off of TMB, the consistency of using different platforms to detect TMB, and the DNA quality assessment methods of biopsy specimens. Thus, screening predictictive biomarkers of ICI efficacy has become the current focus of clinical practice.

Growing evidence shows that specific pathway mutations or specific gene mutations are related to the prognosis of immunotherapy (20, 21). The ZFHX3 mutation is associated with a favorable prognosis for NSCLC receiving ICIs. Studies have shown that the damaged DNA repair mechanism, which results in enhanced immunogenicity and a high mutation load (22). The damaged DNA repair mechanism in patients with NSCLC indicated a sensitive response to PD-1/PD-L1 inhibitors (23). Teo et al. showed that DNA damage response (DDR) pathway mutations may be related to a satisfactory clinical response and significantly prolonged progression-free survival (PFS) and OS in patients with urothelial carcinoma after receiving immunotherapy (21). In addition, Wang et al. showed that comutations in the DDR pathway can be a potential marker for immunotherapy in multiple tumor types (20).

Recently, the immune microenvironment has been discovered to play a vital role in the efficacy of immunotherapy. Studies have shown that tumor-infiltrating lymphocytes (TILs), cytotoxic signatures, and pro-inflammatory mediators are related to favorable immunotherapy efficacy and clinical outcomes (24–26). The past decade has witnessed the importance of a thorough understanding of the cell-intrinsic mechanisms that determine a tumor’s susceptibility to T cell antitumor activity, which was beginning to provide key mechanistic insights into the clinical benefit of potentiating tumor-intrinsic signaling for boosting responses to ICIs (27). The activation of tumor-intrinsic signaling regulates and promotes the immunosuppressive tumor microenvironment, which includes exclusion and dysfunction of effective immunocytes and recruitment and differentiation of immunosuppressive cells (28). TNFα, as a weighted marker of Th1 cells, further mediates antitumor immunity and promotes tumor senescence (29). TNFα promotes the transformation and antitumor functions of TILs and increases the efficacy of ICIs (30). Vredevoogd et al. found that selective reduction of the TNF cytotoxicity threshold increases the susceptibility of tumors to immunotherapy (31). TNF-related apoptosis-inducing ligand (TRAIL) contribute to the antitumor activity of cytotoxic T cells by inducing proliferative arrest and/or apoptosis (32). However, the impact of TNFα-MT on the clinical prognosis of NSCLC patients undergoing immunotherapy is still unclear and needs further exploration. In this study, we mainly analyzed how the mutation status of the TNFα pathway affects the prognosis of ICIs in NSCLC patients from the aspects of tumor immunogenicity and the immune microenvironment.

## Methods

### Clinical Samples

We used the cBioPortal to download mutation data and clinical data from an NSCLC cohort receiving ICIs (33). This cohort, with 344 patients with NSCLC, was defined as the ICI-treated cohort for subsequent analysis. Additionally, we retrospectively collected 36 NSCLC patients (defined as local cohort) from the Zhujiang Hospital of Southern Medical University and performed whole-exome sequencing (WES). Sample preparation, sequencing and raw data processing methods are detailed in the supplementary methods. This study was approved by the Ethics Committee of the Zhujiang Hospital of Southern Medical University, and the patients signed informed consent forms. We used the “TCGAbiolinks” package (34) to download the clinical data, transcription data and mutation data of the TCGA-LUAD and TCGA-LUSC cohorts. We combined the TCGA-LUAD and TCGA-LUSC cohorts into one cohort (TCGA-NSCLC cohort) and used this cohort for downstream analysis. The clinical characteristics of ICI-treated NSCLC, local-NSCLC and TCGA-NSCLC cohort were shown in the **Tables S1**–**S3**.

### Mutation Data Preprocessing and Immunogenicity Data

First, the mutation data were screened with the maftools package (35) according to the nonsynonymous mutation types. Then, we collected the TNFα pathway gene set from the Molecular Signatures Database (MSigDB) (**Table S4**). If the number of mutations in the pathway was 0, then the sample was considered wild type (TNFα-WT); otherwise, it was considered mutant (TNFα-MT). The definitions of TNFα-WT and TNFα-MT were applied to all cohorts in this study. Regarding TMB, TMB score in the ICI-treated cohort was directly obtained from the public data set; in the local cohort and the TCGA-NSCLC cohort, TMB was calculated according to published study. Additionally, the neoantigen load (NAL) and MANTIS scores in the TCGA-NSCLC cohort were reported by previous researchers (36, 37). The DNA damage response (DDR) pathway gene set was obtained from the MSigDB (38). We used the number of nonsynonymous mutations to estimate the number of DDR pathway mutations.

### Immune Microenvironment Analysis and Gene Set Enrichment Analysis

The expression data from the NSCLC cohort and the CIBERSORT algorithm (39) (1000 iterations; parameters: default) were used to evaluate the proportions of twenty-two immune cell types. Additionally, immune-related genes, immune checkpoint-related genes and immune cell fractions were obtained from previous studies. The limma package was used to analyze differences in the expression data of NSCLC patients. After the difference analysis, the data were used as input in the clusterProfiler package (40), and the enrichment scores (ESs) of Gene Ontology (GO) terms, Kyoto Encyclopedia of Genes and Genomes (KEGG) pathways and Reactome pathways were calculated.

### Statistical Analysis

A univariate Cox regression model was used to evaluate the effect of the TNFα pathway and clinical characteristics on the prognosis of patients in the ICI-treated cohort, and hazard ratios (HRs) and 95% confidence intervals (CIs) were used to evaluate their influence. The Wilcoxon rank-sum test was used to compare the differences in continuous variables between the two groups. Fisher’s exact test was used to compare the differences in categorical variables between the two groups. Kaplan-Meier (KM) analysis was used to evaluate the relationship between TNFα-MT and OS, and the log-rank P value was used to reflect significant differences. P <0.05 was considered statistically significant, and all statistical tests were two-sided. R software (version 3.6) was used for statistical analysis.

## Results

### TNFα-MT Is a Predictor of Prolonged Survival for Patients Receiving Immunotherapy

**Figure 1:**
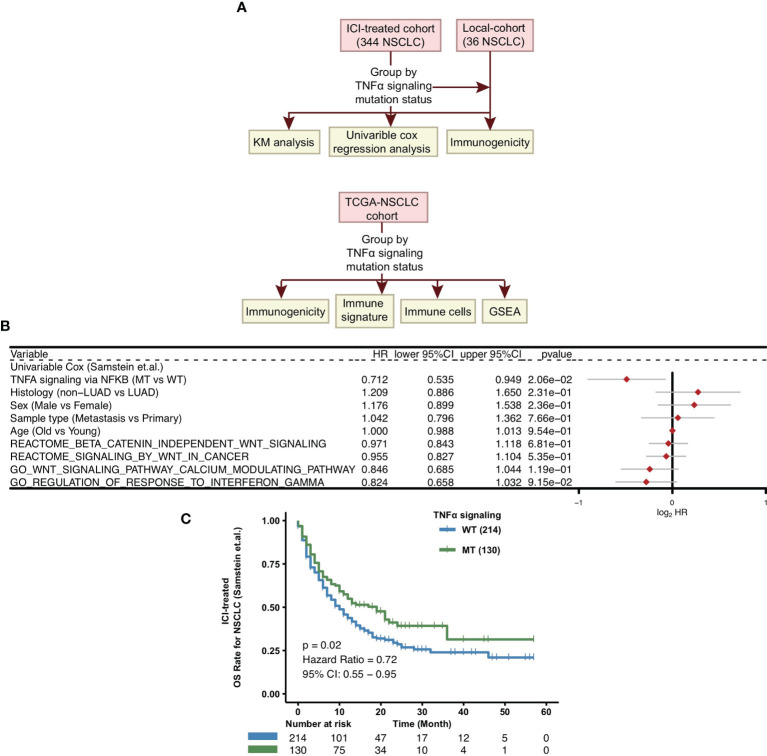
Predictive values of clinical characteristics and the TNFα signaling mutation status on ICI outcomes. **(A)** Flow chart of the establishment of the clinical cohorts and subsequent analyses. **(B)** Forest plot of the results of the univariate Cox regression analyses. **(C)** KM survival curves for OS in NSCLC patients from the ICI-treated cohort. NSCLC, non small-cell lung cancer; TCGA, The Cancer Genome Atlas; ICI, immune checkpoint inhibitors; OS, overall survival; KM, Kaplan Meier.

To explore whether the mutation status of the TNFα pathway can predict the prognosis of patients receiving ICIs for NSCLC, we downloaded the mutation data and survival data of an ICI-treated NSCLC cohort from the cBioPortal website (39). The detailed analysis process is shown in **Figure 1A**. Next, we divided all patients into two groups based on the nonsynonymous mutation status of the TNFα pathway, namely, the TNFα-MT group and the TNFα-WT group. Clinical data, such as age (old *vs*. young), sex (male *vs*. female), histological type (non-LUAD *vs* LUAD), and sample type (metastasis *vs* primary), other pathways (WNT signaling and INFγ singnaling) were not related to the survival of patients in the ICI-treated cohort, but the mutation status of the TNFα pathway was closely associated with the clinical prognosis of patients receiving ICIs (P <0.05; **Figure 1B**). Compared with the TNFα-WT group, the TNFα-MT group had a significantly longer OS (log-rank P = 0.02; HR = 0.72; 95% CI: 0.55-0.95; **Figure 1C**).

### A Panoramic View of Gene Mutations in Different TNFα-MT States

**Figure 2:**
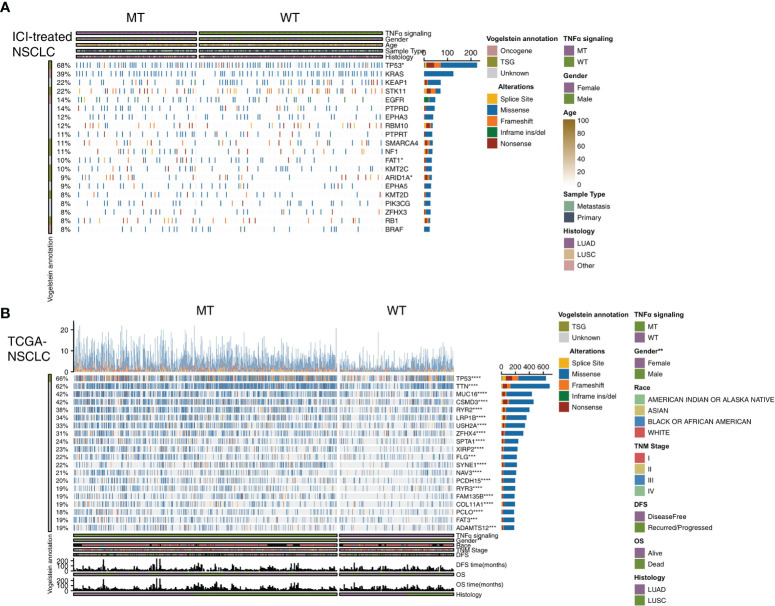
Genomic profiles of NSCLC patients in the ICI-treated **(A)** and TCGA-NSCLC **(B)** cohorts. The top 20 genes with the highest mutation frequencies and the corresponding clinical information are shown in the figure. (*P < 0.05; **P < 0.01; ***P < 0.001; and ****P < 0.0001; Fisher’s exact test). NSCLC, non small-cell lung cancer; TCGA, The Cancer Genome Atlas; ICI, immune checkpoint inhibitors.

To explore the differences in the frequencies of somatic mutations between TNFα-MT and TNFα-WT, we analyzed the top 20 somatic mutations in the ICI-treated cohort and the TCGA-NSCLC cohort. First, in the ICI-treated cohort, we found that among the top 20 mutated genes, the TNFα-MT group had higher mutation rate of TP53 (71% *vs* 58%; P < 0.05), FAT1 (15% *vs* 7%; P < 0.05) and ARID1A (13% *vs* 6%; P < 0.05). Concerning other clinical information, including age, sex, histological type and sample type, there was no significant difference between the TNFα-MT and TNFα-WT groups (**Figure 2A**). Next, we conducted a similar analysis on the TCGA-NSCLC cohort (**Figure 2B**), and the results showed that the TNFα-MT group had a significantly higher frequency of somatic mutations, including all 20 with the highest mutation frequencies (P < 0.05), but the only tumor suppressor gene included in these mutations was TP53. Compared with the TNFα-WT group, the TNFα-MT group had a higher proportion of men (P <0.01). The results of the mutual exclusivity analysis of the top 20 mutated genes in the ICI-treated and TCGA-NSCLC cohorts are shown in **Figure S1**.

### The TNFα-MT Group Has Higher Immunogenicity Than the TNFα-WT Group

**Figure 3:**
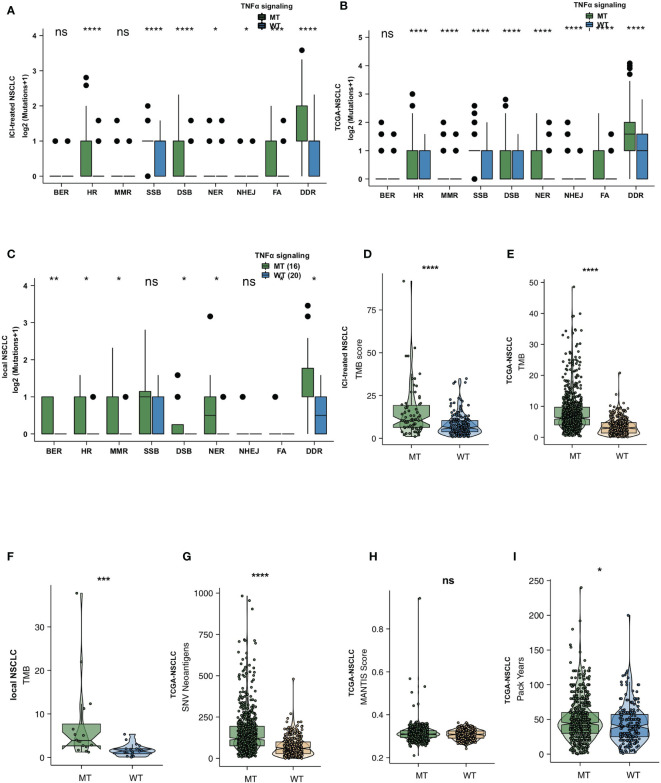
TNFα-MT NSCLC was associated with enhanced tumor immunogenicity. Comparison of DNA damage-related gene set alterations between TNFα-MT and TNFα-WT tumors in the ICI-treated NSCLC **(A)**, TCGA-NSCLC **(B)** and local NSCLC **(C)** cohorts. Comparison of TMB between TNFα-MT and TNFα-WT tumors in the ICI-treated NSCLC **(D)**, TCGA-NSCLC **(E)** and local NSCLC **(F)** cohorts. Comparison of NAL between TNFα-MT and TNFα-WT tumors in the TCGA-NSCLC cohort **(G)**. Comparison of the MANTIS score between TNFα-MT and TNFα-WT tumors in the TCGA-NSCLC cohort **(H)**. Comparison of pack years between TNFα-MT and TNFα-WT tumors in the TCGA-NSCLC cohort **(I)**. (*P < 0.05; **P < 0.01; ***P < 0.001; and ****P < 0.0001; Wilcoxon rank-sum test). NSCLC, non small-cell lung cancer; TCGA, The Cancer Genome Atlas; ICI, immune checkpoint inhibitors. ns, not significant.

To explore the difference between the immunogenicity of the TNFα-MT and TNFα-WT groups, we further elaborated on the number of mutations in the DDR pathway, TMB and NAL. First, we downloaded the gene sets of 8 DDR pathways from the MSigDB and merged all genes related to the DDR pathway into a merged DDR pathway. In the ICI-treated cohort, we found that the TNFα-MT group had a significantly higher number of mutations in the double-strand break (DSB), Fanconi anemia (FA), homologous recombination (HR), nucleotide excision repair (NER), nonhomologous end joining (NHEJ), single-strand break (SSB), and merged DDR pathways than the TNFα-WT group (all P <0.05; **Figure 3A**). In the TCGA-NSCLC cohort, the TNFα-MT group had more mutations in all DDR-related pathways (all P <0.05; **Figure 3B**). Then, we used the local cohort from the Zhujiang Hospital of Southern Medical University for further verification. In the local cohort, we also found that TNFα-MT patients had a higher number of mutations in the DDR pathway (all P <0.05; **Figure 3C**). Additionally, there was a significant difference in DDR signaling mutations according to the mutation status of different TNFα pathways. Regardless of the cohort examined (i.e., the ICI-treated cohort, the TCGA-NSCLC cohort or the local cohort), the TNFα-MT group had a higher TMB than the TNFα-WT group (all P <0.05; **Figures 3D–F**). The TCGA-NSCLC cohort has a significantly high NAL (**Figure 3G**). The MANTIS score can be used to evaluate the microsatellite instability (MSI) status; the higher the score is, the closer its status is to microsatellite instability-high (MSI-H). The MANTIS score of the TNFα-MT group was significantly higher than that of the TNFα-WT group (**Figure 3H**). In addition, the patients in the TNFα-MT group smoked more pack years than those in the TNFα-WT group (P <0.05; **Figure 3I**).

### Differences in Immune Microenvironment Between the TNFα-MT and TNFα-WT Groups

**Figure 4:**
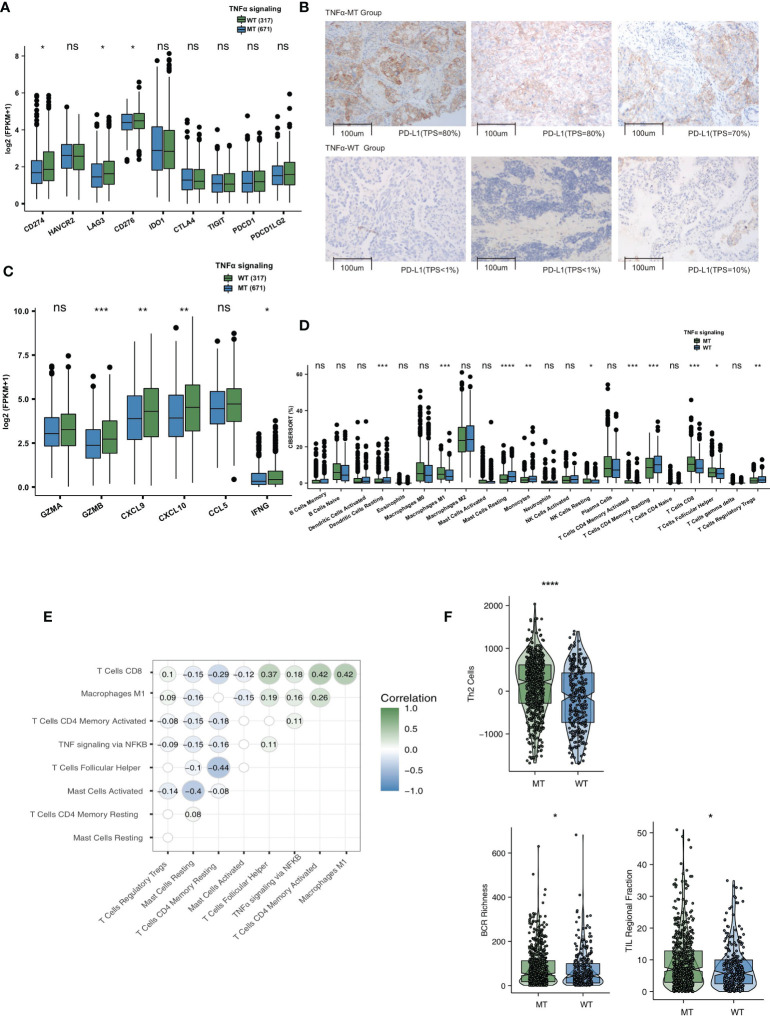
TNFα-MT NSCLC was associated with a significant enrichment of immune cells and enhanced immune scores. **(A)** Comparison of the expression of immune checkpoints between TNFα-MT and TNFα-WT tumors in the TCGA-NSCLC cohort. **(B)** The typical cases for each TPS level between the TNFα-MT (3 samples) and TNFα-WT (3 samples) groups in the Local-NSCLC. **(C)** Comparison of the expression of immune-related genes between TNFα-MT and TNFα-WT tumors in the TCGA-NSCLC cohort. **(D)** Comparison of immune cells between TNFα-MT and TNFα-WT tumors in the TCGA-NSCLC cohort. **(E)** Correlation analysis between the proportions of several immune cell types and number of TNFα signaling mutations. **(F)** Comparison of immune scores between TNFα-MT and TNFα-WT tumors in the TCGA-NSCLC cohort. (*P < 0.05; **P < 0.01; ***P < 0.001; and ****P < 0.0001; Wilcoxon rank-sum test). NSCLC, non small-cell lung cancer; TCGA, The Cancer Genome Atlas; TPS, Tumor Proportion Score. ns, not significant.

To explore the differences in the immune microenvironment between the TNFα-MT and TNFα-WT groups, we compared immune-related genes, immune cell signatures and immune cell types. As the target of ICIs, immune checkpoints are very important in the course of immunotherapy. In the TCGA-NSCLC cohort, we found that the expression levels of PD-L1 (CD274), LAG3 and CD276 were significantly higher in the TNFα-MT group than in the TNFα-WT group (all P <0.05; **Figure 4A**). **Figure 4B** shows typical cases for each TPS level (3 TNFα-MT *vs* 3 TNFα-WT cases). Additionally, some immune-related genes, such as cytotoxicity markers (GZMB), chemokine markers (CXCL9 and CXCL10) and cytokine-related genes (IFNG), were significantly increased in the TNFα-MT group (all P <0.05); **Figure 4C**). At the level of immune cell infiltration, the TNFα-MT group showed a significant enrichment in M1 macrophages, activated memory CD4+ T cells, CD8+ T cells and follicular helper T cells (all P <0.05; **Figure 4D**). Correlation analysis showed that a high number of mutations in TNFα signaling were associated with a high infiltration level of activated immune cells (such as activated memory CD4+ T cells, CD8+ T cells and follicular helper T cells) (R > 0; P <0.05; **Figure 4E**). In contrast, the number of mutations in TNFα signaling was negatively associated with the proportion of Tregs (R <0; P <0.05; **Figure 4E**). The difference analysis of some immune-related signatures showed that the TNFα-MT group had significantly more BCR richness and higher proportions of Th2 cells and TILs than the TNFα-WT group (all P <0.05; **Figure 4F**).

**Figure 5:**
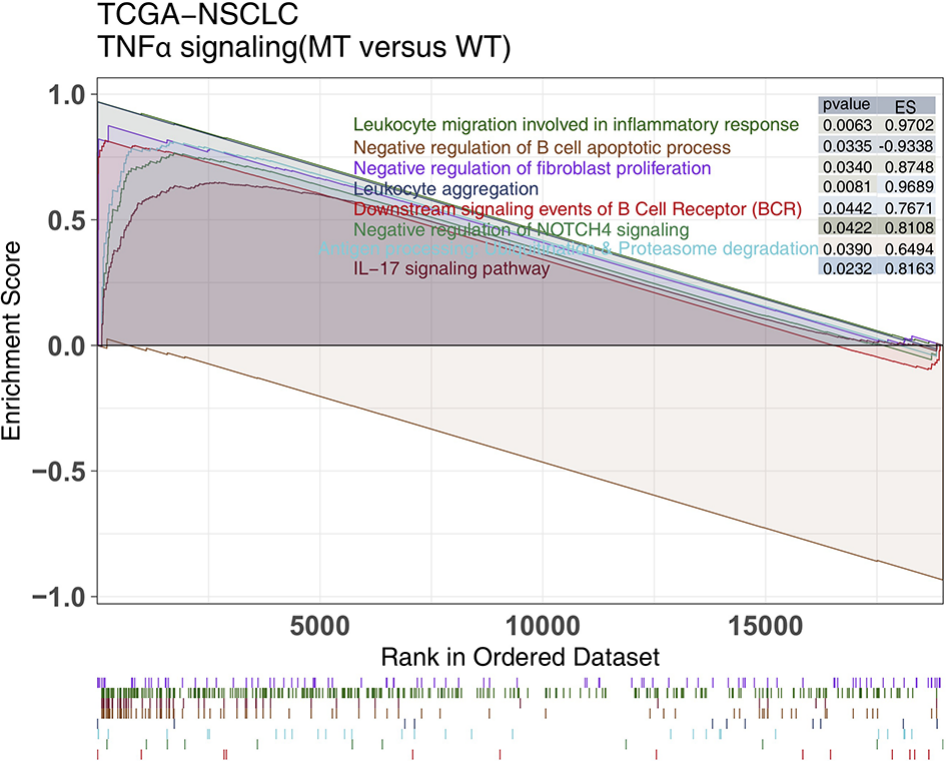
The results of GSEA. The color of the curve corresponds to the font color of the pathway. GSEA of hallmark gene sets downloaded from the MSigDB. Each run was performed with 1000 permutations. Enrichment results with significant differences between TNFα-MT and TNFα-WT tumors are shown. MSigDB, The Molecular Signatures Database; GSEA, Gene Set Enrichment Analysis.

GSEA can be used to examine differences of the enrichment degree of signaling activity between two groups. Therefore, we used GSEA to compare the ESs between the TNFα-MT and TNFα-WT groups. GSEA showed that the activities of immune-related pathways, such as lymphocyte migration activities involved in the inflammatory response, negative regulation of B cell apoptosis, BCR downstream activity, antigen processing and presentation, were significantly increased in the TNFα-MT group (all P <0.05, ES >0; **Figure 5**).

## Discussion

**Figure 6:**
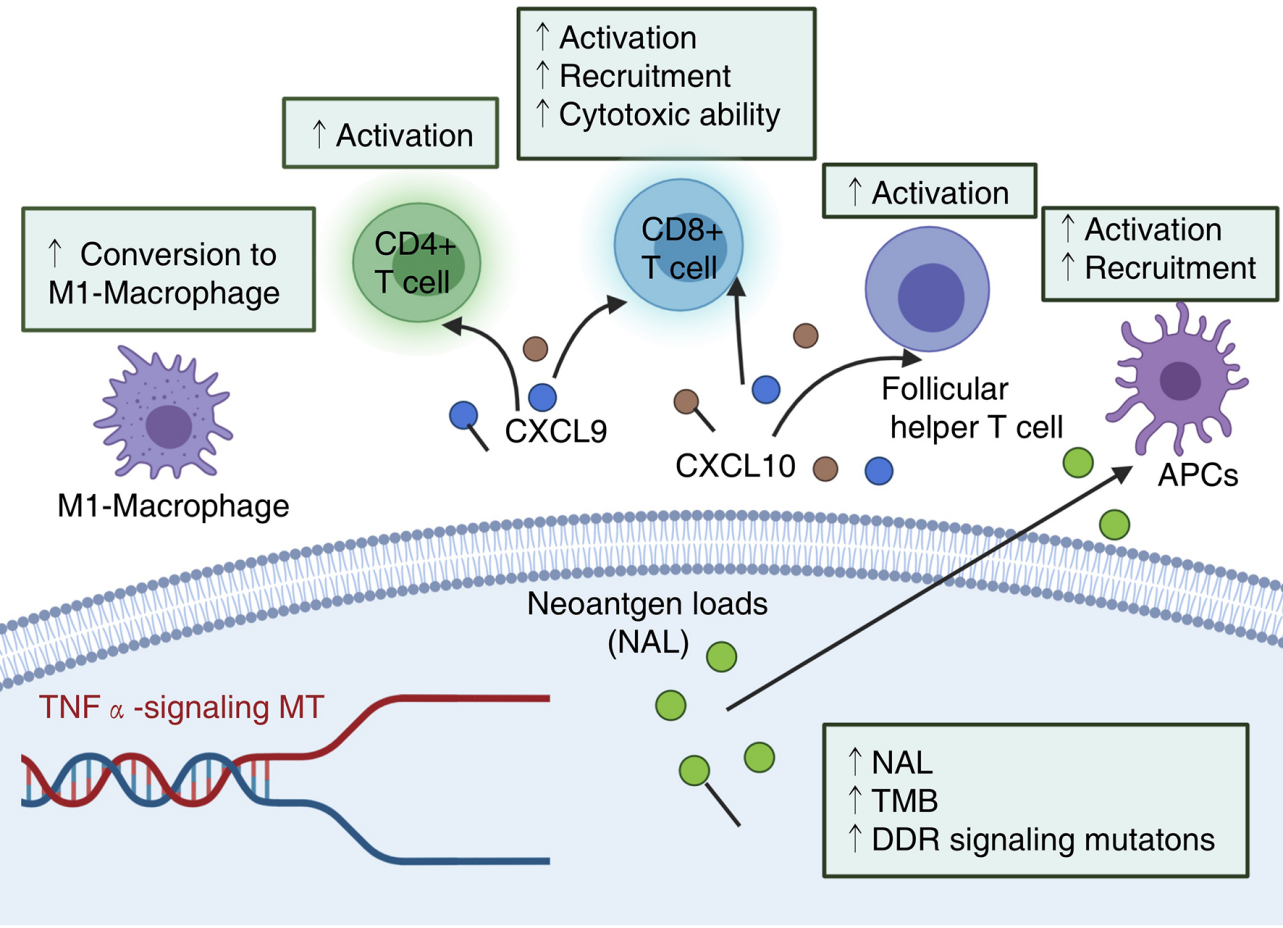
Potential mechanism underlying the prognostic value of TNFα-MT. NAL, neoantigen loads; TMB, Tumor mutational burden; DDR, DNA damage response.

Although ICIs have changed the treatment strategies of NSCLC patients with the development of immunotherapy in recent years, only a small number of patients fully or partially respond to and benefit from ICIs (24, 25, 41). Therefore, for NSCLC patients to better produce an antitumor immune response from ICI treatment and obtain better prognostic outcomes, it is necessary to identify clinically predictive markers. As a stimulatory cytokine, TNFα contributes to the antitumor activity of cytotoxic T cells by inducing proliferative arrest and/or apoptosis, and further enhances tumor cytotoxicity threshold to T cell-derived TNF (31). In this study, we explored the association between TNFα-MT and the prognosis of NSCLC patients receiving ICIs. First, through a univariate Cox regression model and KM analysis, it was found that only TNFα-MT was associated with a favorable prognosis of patients receiving ICIs. Next, we aimed to explain why TNFα-MT was associated with improved clinical benefits in patients from the perspective of the immune microenvironment (**Figure 6**). Patients with TNFα-MT have significantly higher immunogenicity, proportion levels of infiltrating activated immune cells, expression levels of chemokines and cytotoxic markers and MANTIS scores than patients with TNFα-WT. Additionally, we retrospectively collected 36 NSCLC samples from the Zhujiang Hospital of Southern Medical University to further verify the results described above.

ICIs exert an antitumor effect by restoring T cell-mediated antitumor immune function and have become the new clinical treatment approaches for NSCLC. The tumor microenvironment (TME) consists of blood vessels, cancer-associated fibroblasts (CAFs), the extracellular matrix (ECM) and TILs (42). Studies have shown that a local immune imbalance in tumor tissues or tissues surrounding tumors; the systemic immune status, including the number and activity of T cell subsets; antigen recognition, capture, and presentation capabilities; and other host immune stress capabilities also affect immune checkpoints, important aspects that affect the clinical efficacy of inhibitors (19, 24, 25, 41, 43–46).

TILs, especially CD4+ and CD8+ T cells and their immunoregulatory cytokines, play a key role in adaptive immunity. CD8+ T cells produce IFNγ, TNF and granzyme B by binding to T cell receptors and tumor cells, leading to tumor cell clearance (47). However, a variety of such factors have been associated with extrinsic resistance to PD-L1/PD-1 blockade immunotherapy (48). For example, irreversible T cell exhaustion was associated with response or resistance to ICI therapy. Treg cells can directly inhibit the antitumor effect of CD8+ T cells (49). In addition, continuous antigen exposure can cause T cell dysfunction or exhaustion, which is characterized by the loss of effector and memory functions (50). PD-(L)1 inhibitors exert an antitumor effect by reactivating the immune response of T cells to tumors (51). Additionally, studies have found that the baseline status of TILs can also be used as a predictive biomarker for ICI therapy. In a retrospective study of a series of patients (52–56), such as those with colorectal cancer (CRC), melanoma and NSCLC, TILs in tumor biopsy samples were related to favorable OS. Patients with stage III NSCLC receiving immunotherapy have a higher CD8+ TIL density had longer PFS and OS than NSCLC patients a lower CD8+ TILs (57).

In this study, the immune microenvironment of patients with TNFα-MT was significantly enriched in activated memory CD4+ T cells and CD8+ T cells. Tumor-associated macrophages (TAMs) are an important component of immune infiltration in NSCLC. They are highly plastic and exhibit a variety of phenotypes, including the M1 type (classical activation, antitumor activity and proinflammatory response) and the M2 type (nonclassical activation, proangiogenesis and the immunosuppression of original tumor activity) (58). Also, TNF plays a key role in the polarization of macrophages, such as the transformation of myeloid-derived suppressor cells (MDSCs) into M1-like macrophages, which exert antitumor functions (59).

In addition to T cell exhaustion, the release of immunosuppressive cytokines, another extrinsic factor, linked to resistance to ICI therapy (60, 61). However, inflammatory cytokines enriched in the immune microenvironment also play a vital role in the antitumor immune response. For example, chemokines such as CXCL10 and CXCL9 can enhance immune infiltration and antitumor immunity by recruiting CD8+ T cells, dendritic cells (DCs) and natural killer (NK) cells (62). IFNγ can support the proliferation and differentiation of CD8+ T cells (63, 64). Dong et al. demonstrated that IFNγ pretreatment could help CAR-T achieve better therapeutic effects on solid tumors (63). Defects in IFN signal transduction within cancer cells contributed to intrinsic resistance to PD-1 blockade immunotherapy. Gao et al. found that genomic defects in IFNγ pathway genes as primary resistance factor impaired melanoma rejection upon anti-CTLA-4 therapy (60). Additionally, Evgin et al. indicated that type I IFN has negative consequences for CAR T cell viability, and rendering CAR T cells insensitive to type I IFN facilitates combination therapy (62).

The specific immune signature (cytotoxic T lymphocytes signature) is also associated with the prognosis of patients after receiving ICIs (65). Highly expressed cytotoxic markers, such as CD8A, CD8B, GZMA, GZMB and PRF1, are associated with an improved prognosis of immunotherapy (65–67). Recently, CTLA-4, PD-1, TIM-3, TIGIT and other cooperative inhibitory molecules have been shown to be expressed on the surface of immune cells to downregulate immunity, which was another extrinsic resistance factor to ICIs (68). These cells function to protect the host from excessive immune damage. The success of CTLA-4 or PD-1/PD-L1 blockade catalyzed the enthusiasm for a new class of antibody that block negative immune checkpoint regulators for cancer therapy (69). In this study, patients with TNFα-MT had higher expression levels of immune checkpoints, such as PD-L1 (CD274), LAG3 and CD276, than patients with TNFα-WT.

Tumor immunogenicity has also been shown to be related to the efficacy of immunotherapy, which can be assessed *via* TMB, NAL, MSI-H, DDR pathway mutations and antigen processing and presentation signatures (70–73). Insufficient tumor antigenicity was another intrinsic factor contributing to immunoresistance (74). Alterations in the DDR pathway may lead to the accumulation of uncorrected DNA damage and ultimately increase tumor immunogenicity (18, 44, 75, 76). In this study, we found that patients with TNFα-MT had higher immunogenicity, which was manifested as an upregulated TMB and NAL. The MANTIS score can also be used to evaluate the MSI score. The higher the score is, the closer its status is to MSI-H. The MANTIS score of the TNFα-MT group was significantly higher than that of the TNFα-WT group. Based on the results described above, we believe that the upregulated immunogenicity in the TNFα-MT group may represent one of the potential factors that results in these patients having a satisfactory clinical prognosis after receiving immunotherapy (77).

Although this study, from the perspective of the immune microenvironment (i.e., immune cells, immune-related signatures, immunogenicity, and cytokines) explored the impact of TNFα signaling mutations on the prognosis of NSCLC patients receiving ICIs, there are still some limitations. First, we only analyzed a cohort of patients receiving immunotherapy; therefore, we hope to recruit more NSCLC patients receiving immunotherapy for follow-up verification. Second, in the ICI-treated cohort, only the targeted sequencing data were analyzed; this mutation data were far less than those of WES, and transcriptome, proteomics and other genomic data were lacking. Third, we used only the TCGA-NSCLC cohort and a local cohort containing 36 NSCLC patients from the Zhujiang Hospital of Southern Medical University for verification. Fourth, we did not perform related cell experiments or animal experiments to directly prove our hypothesis; corresponding cell experiments and animal experiments will be done in the future. Fifth, TNF-MT signature may indeed be a mirror of a T-cell cytotoxicity signature, but this is only a hypothesis, because we are more to elaborate the correlation between TNF-MT signature and TIME. We hope that we can further explore association between the T-cell cytotoxicity signature and prognosis of immunotherapy. Finally, we hope that we can collect more cancer types to validate the role of TNFα signaling on the prognosis related to immunotherapy.

## Conclusions

In this study, compared with TNFα-WT NSCLC, TNFα-MT NSCLC had a better prognosis for immunotherapy. Additionally, we found that TNFα-MT showed a significant enrichment in activated immune cells, upregulated immunogenicity and increased immune-related signatures. Therefore, TNFα-MT may serve as potential biomarkers for clinically guiding NSCLC patients to receive immunotherapy.

## Data Availability Statement

The datasets presented in this study can be found in online repositories. The names of the repository/repositories and accession number(s) can be found in the article/**Supplementary Material**.

## Ethics Statement

The patients/participants provided their written informed consent to participate in this study and the research presented here has been performed in accordance with the Declaration of Helsinki and has been approved by the ethics committee of the Zhujiang Hospital of Southern Medical University.

## Author Contributions

Writing-original draft, AL. Conceptualization, PL and JZ. Investigation, AL. Writing-review and editing, AL, HZ, HM, PL and JZ. Formal analysis, AL, HZ and HM. Visualization, AL, HZ, HM, ZD and TG. All authors contributed to the article and approved the submitted version.

## Funding

This work was supported by the Natural Science Foundation of Guangdong Province (2018A030313846, 2021A1515012593), the Science and Technology Planning Project of Guangdong Province (2019A030317020) and the National Natural Science Foundation of China (81802257, 81871859, 81772457).

## Conflict of Interest

The authors declare that the research was conducted in the absence of any commercial or financial relationships that could be construed as a potential conflict of interest.

## Publisher’s Note

All claims expressed in this article are solely those of the authors and do not necessarily represent those of their affiliated organizations, or those of the publisher, the editors and the reviewers. Any product that may be evaluated in this article, or claim that may be made by its manufacturer, is not guaranteed or endorsed by the publisher.

## Supplementary Material

Supplementary Figure 1 Results of the cooccurrence/mutual exclusivity analysis of the top 20 mutated genes in the ICI-treated NSCLC **(A)** and TCGA-NSCLC **(B)** cohorts.

Supplementary Table 1 Baseline characteristics of NSCLC patients (ICI-treated NSCLC cohort).

Supplementary Table 2 Baseline characteristics of NSCLC patients (TCGA-NSCLC cohort).

Supplementary Table 3 Baseline characteristics of NSCLC patients (Local-NSCLC cohrot).

Supplementary Table 4 The TNFα pathway gene set from the Molecular Signatures Database (MSigDB).
