# Supplementary figures and images for: TNF-Alpha Pathway Alternation Predicts Survival of Immune Checkpoint Inhibitors in Non-Small Cell Lung Cancer
Source: Front Immunol. 2021 Sep 16;12:667875. doi: 10.3389/fimmu.2021.667875 (PMC8481577; doi:10.3389/fimmu.2021.667875)

**a**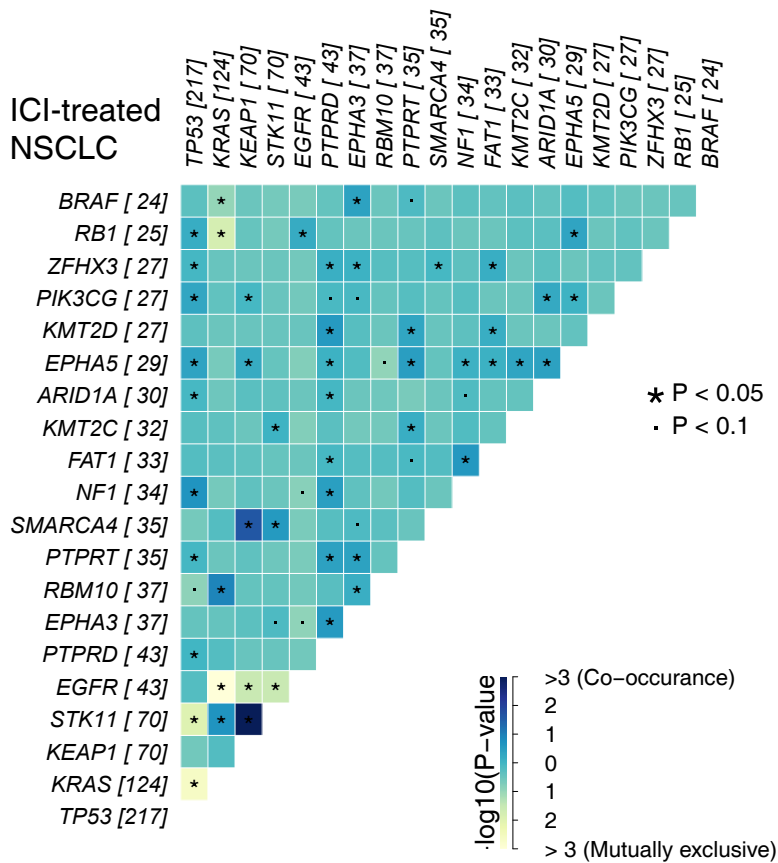**b**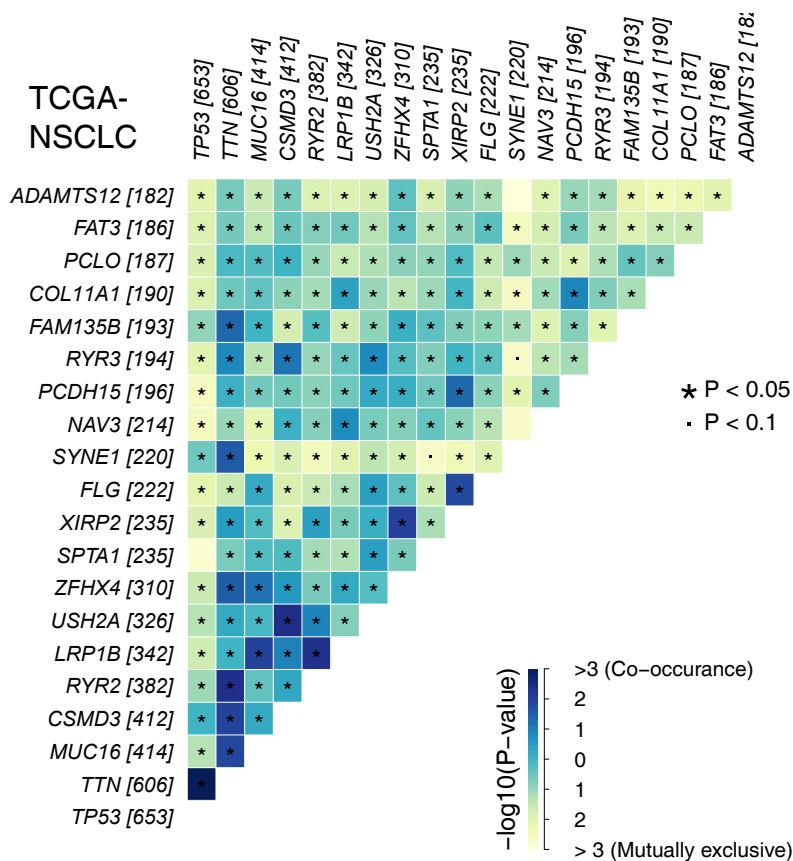

Supplement: Supplementary Figure 1 — Results of the cooccurrence/mutual exclusivity analysis of the top 20 mutated genes in the ICI-treated NSCLC (A) and TCGA-NSCLC (B) cohorts. [file Image_1.pdf]
