# Supplementary material for: TNF-Alpha Pathway Alternation Predicts Survival of Immune Checkpoint Inhibitors in Non-Small Cell Lung Cancer
Source: Front Immunol. 2021 Sep 16;12:667875. doi: 10.3389/fimmu.2021.667875 (PMC8481577; doi:10.3389/fimmu.2021.667875)
Supplement: Supplementary Table 1 — Baseline characteristics of NSCLC patients (ICI-treated NSCLC cohort). [file Table_1.pdf]

	TNFα-WT (N=214)	TNFα-MT (N=130)	Overall (N=344)
**Gender**			
Female	113 (52.8%)	65 (50.0%)	178 (51.7%)
Male	101 (47.2%)	65 (50.0%)	166 (48.3%)
**Sample Type**			
Metastasis	109 (50.9%)	67 (51.5%)	176 (51.2%)
Primary	105 (49.1%)	63 (48.5%)	168 (48.8%)
**Histology**			
LUAD	174 (81.3%)	92 (70.8%)	266 (77.3%)
non-LUAD	40 (18.7%)	38 (29.2%)	78 (22.7%)
**OS**			
Alive	69 (32.2%)	61 (46.9%)	130 (37.8%)
Dead	145 (67.8%)	69 (53.1%)	214 (62.2%)
**Age**			
Mean (SD)	65.2 (10.7)	66.6 (10.7)	65.8 (10.7)
Median [Min, Max]	67.0 [31.0, 89.0]	67.0 [32.0, 90.0]	67.0 [31.0, 90.0]
**OS time**			
Mean (SD)	12.4 (12.4)	13.2 (10.9)	12.7 (11.9)
Median [Min, Max]	9.00 [0, 57.0]	12.0 [0, 57.0]	10.0 [0, 57.0]
