# Supplementary material for: TNF-Alpha Pathway Alternation Predicts Survival of Immune Checkpoint Inhibitors in Non-Small Cell Lung Cancer
Source: Front Immunol. 2021 Sep 16;12:667875. doi: 10.3389/fimmu.2021.667875 (PMC8481577; doi:10.3389/fimmu.2021.667875)
Supplement: Supplementary Table 2 — Baseline characteristics of NSCLC patients (TCGA-NSCLC cohort). [file Table_2.pdf]

	TNFα-WT (N=317)	TNFα-MT (N=671)	Overall (N=988)
**Gender**			
Female	150 (47.3%)	247 (36.8%)	397 (40.2%)
Male	167 (52.7%)	424 (63.2%)	591 (59.8%)
**Histology**			
LUAD	192 (60.6%)	313 (46.6%)	505 (51.1%)
LUSC	125 (39.4%)	358 (53.4%)	483 (48.9%)
**Race**			
AMERICAN INDIAN OR ALASKA NATIVE	0 (0%)	1 (0.1%)	1 (0.1%)
ASIAN	4 (1.3%)	12 (1.8%)	16 (1.6%)
BLACK OR AFRICAN AMERICAN	24 (7.6%)	55 (8.2%)	79 (8.0%)
WHITE	234 (73.8%)	486 (72.4%)	720 (72.9%)
Missing	55 (17.4%)	117 (17.4%)	172 (17.4%)
**OS**			
Alive	187 (59.0%)	408 (60.8%)	595 (60.2%)
Dead	130 (41.0%)	263 (39.2%)	393 (39.8%)
**Pack Years**			
Mean (SD)	43.3 (27.1)	49.0 (30.2)	47.4 (29.4)
Median [Min, Max]	40.0 [1.00, 200]	42.0 [0.150, 240]	40.0 [0.150, 240]
Missing	97 (30.6%)	134 (20.0%)	231 (23.4%)
**OS time**			
Mean (SD)	30.2 (27.1)	30.9 (31.8)	30.7 (30.4)
Median [Min, Max]	21.6 [0, 164]	21.4 [0, 238]	21.5 [0, 238]
Missing	7 (2.2%)	8 (1.2%)	15 (1.5%)
