# Supplementary material for: TNF-Alpha Pathway Alternation Predicts Survival of Immune Checkpoint Inhibitors in Non-Small Cell Lung Cancer
Source: Front Immunol. 2021 Sep 16;12:667875. doi: 10.3389/fimmu.2021.667875 (PMC8481577; doi:10.3389/fimmu.2021.667875)
Supplement: Supplementary Table 3 — Baseline characteristics of NSCLC patients (Local-NSCLC cohrot). [file Table_3.pdf]

	TNFα-WT (N=20)	TNFα-MT (N=16)	Overall (N=36)
**Age**			
Mean (SD)	58.2 (11.1)	56.7 (11.7)	57.7 (11.2)
Median [Min, Max]	59.5 [41.0, 78.0]	55.0 [39.0, 75.0]	58.0 [39.0, 78.0]
Missing	0 (0%)	5 (31.2%)	5 (13.9%)
