# Supplementary material for: TNF-Alpha Pathway Alternation Predicts Survival of Immune Checkpoint Inhibitors in Non-Small Cell Lung Cancer
Source: Front Immunol. 2021 Sep 16;12:667875. doi: 10.3389/fimmu.2021.667875 (PMC8481577; doi:10.3389/fimmu.2021.667875)
Supplement: Supplementary Table 4 — The TNFα pathway gene set from the Molecular Signatures Database (MSigDB). [file Table_4.pdf]

HALLMARK_TNFA_SIGNALING_VIA_NFKB
ABCA1
ACKR3
AREG
ATF3
ATP2B1
B4GALT1
B4GALT5
BCL2A1
BCL3
BCL6
BHLHE40
BIRC2
BIRC3
BMP2
BTG1
BTG2
BTG3
CCL2
CCL20
CCL4
CCL5
CCN1
CCND1
CCNL1
CCRL2
CD44
CD69
CD80
CD83
CDKN1A
CEBPB
CEBPD
CFLAR
CLCF1
CSF1
CSF2
CXCL1
CXCL10
CXCL11

CXCL2
CXCL3
CXCL6
DDX58
DENND5A
DNAJB4
DRAM1
DUSP1
DUSP2
DUSP4
DUSP5
EDN1
EFNA1
EGR1
EGR2
EGR3
EHD1
EIF1
ETS2
F2RL1
F3
FJX1
FOS
FOSB
FOSL1
FOSL2
FUT4
G0S2
GADD45A
GADD45B
GCH1
GEM
GFPT2
GPR183
HBEGF
HES1
ICAM1
ICOSLG
ID2
IER2

IER3
IER5
IFIH1
IFIT2
IFNGR2
IL12B
IL15RA
IL18
IL1A
IL1B
IL23A
IL6
IL6ST
IL7R
INHBA
IRF1
IRS2
JAG1
JUN
JUNB
KDM6B
KLF10
KLF2
KLF4
KLF6
KLF9
KYNU
LAMB3
LDLR
LIF
LITAF
MAFF
MAP2K3
MAP3K8
MARCKS
MCL1
MSC
MXD1
MYC
NAMPT

NFAT5
NFE2L2
NFIL3
NFKB1
NFKB2
NFKBIA
NFKBIE
NINJ1
NR4A1
NR4A2
NR4A3
OLR1
PANX1
PDE4B
PDLIM5
PER1
PFKFB3
PHLDA1
PHLDA2
PLAU
PLAUR
PLEK
PLK2
PLPP3
PMEPA1
PNRC1
PPP1R15A
PTGER4
PTGS2
PTPRE
PTX3
RCAN1
REL
RELA
RELB
RHOB
RIPK2
RNF19B
SAT1
SDC4

SERPINB2
SERPINB8
SERPINE1
SGK1
SIK1
SLC16A6
SLC2A3
SLC2A6
SMAD3
SNN
SOCS3
SOD2
SPHK1
SPSB1
SQSTM1
STAT5A
TANK
TAP1
TGIF1
TIPARP
TLR2
TNC
TNF
TNFAIP2
TNFAIP3
TNFAIP6
TNFAIP8
TNFRSF9
TNFSF9
TNIP1
TNIP2
TRAF1
TRIB1
TRIP10
TSC22D1
TUBB2A
VEGFA
YRDC
ZBTB10
ZC3H12A

ZFP36
