# Supplementary material for: TNF-Alpha Pathway Alternation Predicts Survival of Immune Checkpoint Inhibitors in Non-Small Cell Lung Cancer
Source: Front Immunol. 2021 Sep 16;12:667875. doi: 10.3389/fimmu.2021.667875 (PMC8481577; doi:10.3389/fimmu.2021.667875)
Supplement: Supplementary file 6 [file DataSheet_1.docx]

Supplementary Methods

Human non-small cell lung cancer specimens

The study protocol was approved by the Ethics Committee of the Zhujiang Hospital of Southern Medical University, Guangzhou, China, and informed consent was obtained for all studies. Informed consent (in writing) was obtained from each patient, and the study protocol conformed to the ethical guidelines of the 1975 Declaration of Helsinki as reflected in a prior approval by the Ethics Committee of the Zhujiang Hospital of Southern Medical University, Guangzhou, China. The diagnosis of NSCLC was made by pathologists in the above hospitals by hematoxylin and eosin (H&E) staining according to histology plus immunohistochemistry for chromogranin A and synaptophysin. Of the 36 patients, 36 formalin-fixed paraffin-embedded (FFPE) tumor samples had matched germline specimens (n=36). WES was performed on 36 NSCLC FFPE tumor samples and matched germline specimens.

Whole-exome sequencing (WES) and data processing

DNA was extracted from each FFPE specimen (n=36) and matched germline specimen (n=36) using a Gentra Puregene DNA Extraction Kit (Qiagen) following the protocol of the manufacturer. All sequencing data underwent quality control for read counts, quality values, GC content, and all other relevant parameters with FastQC (v0.10.1). The raw paired sequencing reads of human samples acquired from WES were aligned to the respective human genome build (hg38). Alignment was performed with the Burrows-Wheeler aligner[1] (version 0.6.1-r104). Concordant read pairs were identified as potential PCR duplicates and were subsequently masked in the alignment file. Additionally, an estimation of human DNA library contamination was implemented to enhance the sensitivity and specificity of mutation calling. SAMtools mpileup (0.1.19)[2] was used to locate nonreference positions in the tumor and germline samples. After removing terminal adaptor sequences and low-quality data, reads were mapped to the reference human genome (hg38) and aligned using BWA (version 0.6.1-r104). MuTect2 (3.4–46-gbc02625)[3] was employed to call somatic small insertions and deletions (InDels) and single nucleotide variants (SNVs).

Pathological diagnosis

Pathological diagnosis was conducted by pathologist using hematoxylineosin (HE) stained slides. After the diagnosis of NSCLC, PD-L1 immunohistochemistry staining of each biopsy sample was conducted and assessed by at least two pathologists. The samples, which were formalin-fixed and paraffin-embedded, were sliced at a thickness of 4 μm. The sections were processed for 20 min at 97°C for deparaffinized and inactivating enzymes. Sequentially, the samples were stained for PD-L1 with an anti-human PD-L1 antibody. PD-L1 expression was evaluated in our institution using companion diagnostic PD-L1 immunohistochemistry (IHC) (PD-L1 IHC 22C3, pharmDx, Dako/Agilent, Santa Clara, United States) with autostainer Link 48, detecting driver mutation in parallel. Following the standard recommendation of previous publication[4] PD-L1 protein expression was determined by tumor proportion score (TPS).

1. Li, H.; Durbin, R. Fast and accurate short read alignment with Burrows-Wheeler transform. *Bioinformatics* **2009**, *25*, 1754–1760, doi:10.1093/bioinformatics/btp324.

2. Li, H.; Handsaker, B.; Wysoker, A.; Fennell, T.; Ruan, J.; Homer, N.; Marth, G.; Abecasis, G.; Durbin, R. The Sequence Alignment/Map format and SAMtools. *Bioinformatics* **2009**, *25*, 2078–2079, doi:10.1093/bioinformatics/btp352.

3. Cibulskis, K.; Lawrence, M.S.; Carter, S.L.; Sivachenko, A.; Jaffe, D.; Sougnez, C.; Gabriel, S.; Meyerson, M.; Lander, E.S.; Getz, G. Sensitive detection of somatic point mutations in impure and heterogeneous cancer samples. *Nat. Biotechnol.* **2013**, *31*, 213–219, doi:10.1038/nbt.2514.

4 Tsunoda A, Morikawa K, Inoue T, Miyazawa T, Hoshikawa M, Takagi M, Mineshita M. A prospective observational study to assess PD-L1 expression in small biopsy samples for non-small-cell lung cancer. BMC cancer. 2019 Dec;19(1):1-8.
